# Benzodiazepine prescribing in chronic military-related PTSD: real-world patterns and correlates in specialized care

**DOI:** 10.3389/fpsyt.2026.1931305

**Published:** 2026-10-05

**Authors:** Erin Collins, Jenny J. W. Liu, Mathieu Bilodeau, Amer M. Burhan, Murray W. Enns, Vicky Lavoie, Natalie Ein, Anthony Nazarov, Edouard Auger, Ken Balderson, Natalie Mota, Sonya Wanklyn, J. Don Richardson

**Affiliations:** 1 MacDonald Franklin OSI Research Centre, Lawson Research Institute, London, ON, Canada; 2 Department of Psychiatry, Western University, London, ON, Canada; 3 Clinique TSO du CIUSSS de la Capitale Nationale, Québec, QC, Canada; 4 Département de Psychiatrie et de Neurosciences, Faculté de Médecine, Université Laval, Québec, QC, Canada; 5 Department of Psychiatry, University of Toronto, Toronto, ON, Canada; 6 Ontario Shores Centre of Mental Health Sciences, Whitby, ON, Canada; 7 Department of Psychiatry, Max Rady College of Medicine, University of Manitoba, Winnipeg, MB, Canada; 8 Deer Lodge Centre OSI Clinic, Winnipeg, MB, Canada; 9 Department of Psychiatry and Behavioural Neurosciences, McMaster University, Hamilton, ON, Canada; 10 St. Joseph’s OSI Clinic, St. Joseph’s Health Care London, London, ON, Canada; 11 Department of Clinical Health Psychology and Psychiatry, University of Manitoba, Winnipeg, MB, Canada; 12 Lawson Research Institute, London, ON, Canada

**Keywords:** benzodiazepine, military, operational stress injury (OSI), pharmacotherapy, psychotropics, PTSD - posttraumatic stress disorder, veteran

## Abstract

**Objective:**

To estimate the prevalence of benzodiazepine prescribing among patients with posttraumatic stress disorder (PTSD), identify patient- and practice-level correlates of benzodiazepine use, and examine the clinical rationale underlying prescribing decisions. We aimed to characterize real-world patterns and determinants of benzodiazepine use in a specialized tertiary care military and Veteran PTSD setting, where PTSD is often chronic and treatment-refractory, and to clarify the clinical factors associated with benzodiazepine prescribing.

**Methods:**

We conducted a cross-sectional study using clinician-reported data from 13 psychiatrists practicing in Canadian tertiary care Operational Stress Injury clinics. Psychiatrists provided current prescribing information for 438 military personnel and Veterans receiving treatment for a primary diagnosis of PTSD. Multivariable logistic regression examined associations between patient and provider characteristics, treatment history, and benzodiazepine prescribing. Analyses adjusted for age, sex, and clinical site, and accounted for clustering at the provider level.

**Results:**

Benzodiazepines were prescribed to 22% of patients. Use was more frequent among patients with longer PTSD duration and greater cumulative pharmacotherapy exposure. Each additional year of PTSD was associated with a 12% increase in adjusted odds of benzodiazepine use (OR 1.12, 95% CI 1.06–1.18). Patients with ≥ 5 years of pharmacotherapy exposure had two- to three-fold greater odds compared with those treated < 5 years. Benzodiazepine monotherapy was rare (< 1%), with most use occurring in combination with other psychotropic medications.

**Conclusions:**

In these specialized clinics, benzodiazepine use was more common among patients with persistent illness and extensive treatment histories. These patterns suggest use occurs primarily in the context of chronic and complex care, underscoring the importance of clear clinical guidance for real-world practice.

## Introduction

Benzodiazepines are generally discouraged in the treatment of posttraumatic stress disorder (PTSD). Major clinical practice guidelines, including the 2023 U.S. Department of Veterans Affairs and Department of Defense (VA/DoD) guideline (1) and the National Institute for Health and Care Excellence (NICE) (2), recommend against their routine use, citing limited evidence for improvement in core PTSD symptoms and concerns regarding tolerance, dependence, cognitive adverse effects, and potential interference with trauma-focused psychotherapy (3–13). Randomized controlled trials have not demonstrated consistent benefit across PTSD outcomes (7, 14, 15), and observational studies have raised concerns regarding misuse, overdose risk, and suicidality (9, 11, 16–23).

Despite these recommendations, benzodiazepines continue to be prescribed in clinical practice, including in military and Veteran populations (24). Military-related PTSD is frequently chronic and complex, characterized by prolonged symptom duration, multimorbidity, and high rates of prior treatment exposure (25–28). Clinicians in these settings may encounter patients with persistent insomnia, hyperarousal, and anxiety despite adequate trials of trauma-focused psychotherapy and first-line pharmacotherapies (25, 26, 29, 30). For some individuals, symptoms remain severe and functionally impairing even after multiple evidence-based interventions.

How often benzodiazepines are prescribed under these circumstances is difficult to summarize, as reported prevalence varies across populations, settings, and eras. Within the U.S. Veterans Health Administration (VHA), benzodiazepine prescribing among patients with PTSD declined from 31.3% in 2009 to 10.7% in 2019 (31). Two U.S. studies report estimates in the 20% to 25% range: one among active-duty service members with a diagnosis of PTSD between 2007 and 2013 (32), and one among patients with primary PTSD diagnosed in a large health system between 2002 and 2011 (33). Higher estimates have been reported in more acute populations, such as 47% among Australian Veterans who were taking benzodiazepines at the time of admission for inpatient PTSD care in 2019 to 2020 (34). In Canada, data remain sparse: 31.2% of a Quebec sample of older adults seeking primary care between 2011 and 2013 had at least one benzodiazepine dispensed in the preceding 6 months, and probable post-traumatic stress syndrome was associated with greater odds of use (35). Benzodiazepine prescribing has not previously been characterized within specialized Canadian PTSD care.

Where prescribing does occur, it takes place within a broader clinical and organizational landscape. Clinician training, familiarity with psychopharmacology, and perceptions regarding the applicability of civilian-derived guidelines to military populations may influence practice patterns (26, 36–39). Additional considerations include the limited number of approved pharmacotherapies for PTSD and the delayed onset of antidepressant benefit, both of which may restrict immediate treatment options for patients in distress.

Together, these multi-level influences may help explain the persistence of benzodiazepine prescribing in military-related PTSD. However, little empirical work has examined how these medications are used within specialized military and Veteran PTSD clinics, or which clinical and practice-level factors are associated with their prescription. Without such data, prescribing patterns may be viewed as arbitrary without consideration of the clinical context in which decisions are made.

The present study examines benzodiazepine prescribing among Canadian military personnel and Veterans receiving care for primary PTSD in Operational Stress Injury (OSI) clinics. Specifically, we sought to: (1) estimate the prevalence of benzodiazepine prescribing for PTSD; (2) identify patient- and practice-level correlates of benzodiazepine use; and (3) situate these findings within a clinical interpretation of prescribing rationale. By characterizing real-world prescribing patterns in a specialized military and Veteran population, in which PTSD is often chronic and treatment-refractory, this study aims to clarify the clinical and contextual factors that may influence benzodiazepine use.

## Materials and methods

### Study design and data source

This study utilized data from a survey of psychiatrists treating PTSD within OSI clinics, which provide specialized mental health care to military members, Veterans, and public safety personnel. Details of the survey methodology, sampling strategy, and instrument development have been described previously (24). Ethics and institutional approvals were obtained from the Western University Research Ethics Board (WREM #119200) and the Lawson Health Research Institute (REDA #11319), respectively. Study procedures were developed in collaboration with the MIND PTSD Collaborative, a national interdisciplinary panel of psychiatrists, psychologists, and researchers with expertise in CAF (Canadian Armed Forces) and Royal Canadian Mounted Police (RCMP) trauma-related mental health care. Reporting adheres to the Strengthening the Reporting of Observational Studies in Epidemiology (STROBE) guidelines (40).

### Participants and sampling

Thirteen psychiatrists practicing in OSI clinics across three regions (Toronto/London, Winnipeg, and Quebec/Saguenay) completed the survey. For each psychiatrist, data were collected on provider characteristics, assessment practices, and the factors they considered important when prescribing pharmacotherapy for PTSD. Participating psychiatrists then reported prescribing information for patients in their current caseload who were current or former members of the CAF and/or the RCMP and who had a primary clinical diagnosis of PTSD. This process yielded clinician-reported treatment data for 438 patients, which constitute the analytic sample. Currently serving and Veteran members of the RCMP accounted for < 10% of the sample. For simplicity, we refer to all patients as “military and Veteran” regardless of most recent service affiliation (CAF or RCMP).

### Measures

#### Outcome

The primary outcome was clinician-reported benzodiazepine prescribing, defined as the presence or absence of at least one benzodiazepine prescription. For each psychotropic medication, participating psychiatrists indicated whether the prescription was intended specifically to manage symptoms of PTSD. Benzodiazepines were identified using pharmacologic classifications from *UpToDate* and *Medscape*, and final categorizations were confirmed by consensus with the MIND PTSD Collaborative. The following benzodiazepines were present in the dataset: lorazepam, clonazepam, temazepam, flurazepam, and diazepam. Other classes of hypnotic agents (e.g., trazodone, zopiclone, zolpidem, eszopiclone, lemborexant, doxepin) were classified separately. A comprehensive list of all psychotropic agents and classifications is provided in the primary publication (24).

#### Predictors and covariates

Pre-specified predictors and covariates included patient-level and practice-level factors, selected based on prior literature and their clinical relevance to benzodiazepine prescribing in PTSD. Patient-level variables encompassed demographics (age, sex), service status (Veteran vs active duty), comorbidities, PTSD chronicity (years since diagnosis), and treatment exposure, including duration of pharmacotherapy for PTSD (<5, 5–10, >10 years), current receipt of any psychotherapy (yes/no), and receipt of psychiatrist-rated evidence-based psychotherapy (yes/no). Practice-level variables included clinical site (Winnipeg, Toronto/London, or Quebec/Saguenay) to account for regional and organizational differences in service delivery, and provider, to account for potential heterogeneity in prescribing practices.

### Analysis

Descriptive statistics were used to characterize the sample and to compare patients who received benzodiazepines with those who did not. As psychiatrists specified whether each medication was prescribed to manage PTSD specifically, analyses were conducted under two definitions: (1) benzodiazepine prescribing for PTSD vs no benzodiazepine prescribing for PTSD, and (2) benzodiazepine prescribing for any indication (including PTSD) vs no benzodiazepine prescribing for any indication. Group differences were examined using statistical tests appropriate to variable type and distribution. Continuous variables were compared using independent-samples *t* tests when normality assumptions were met, and Mann–Whitney *U* tests otherwise. Categorical variables were compared using χ² tests or Fisher exact tests when expected cell counts were <5.

Associations between *a priori* predictors and benzodiazepine prescribing were examined using logistic regression. Multivariable models controlled for age, sex, and study site and included provider-level random effects to account for clustering by prescriber. Variables with >5% missingness were imputed using multiple imputation by chained equations, contingent on the data meeting a missing-at-random assumption. All analyses were conducted using R (version 4.5.1).

## Results

The cohort of patients (*n* = 438) was predominantly male (88%) and Veteran (90%), with a mean age of 47.7 years (SD = 9). PTSD had been diagnosed for an average of 8.2 years (SD = 5.3), and long-term pharmacotherapy was common, with 63% (*n* = 274) receiving medication for ≥ 5 years.

### Demographics and military characteristics by benzodiazepine prescribing status

**Table 1** summarizes demographic, health, and military characteristics stratified by benzodiazepine prescribing status for PTSD (yes: *n* = 98; no: *n* = 340) and for any indication (yes: *n* = 118; no: *n* = 320). Site-level differences were significant for the “any indication” comparison (*p* < 0.01), but not for the PTSD-specific comparison (*p* = 0.07). Physical and mental health comorbidities did not differ substantially between strata and were highly prevalent overall (>75%), reflecting the clinical complexity characteristic of this population.

**Table 1:** Characteristics of patients with a primary PTSD diagnosis (*n* = 438) by benzodiazepine prescription status^†^ for PTSD and for any indication.

Patient characteristic	Benzodiazepines prescribed for PTSD		*p*-value	Benzodiazepines prescribed for any indication		
	No (*n* = 340)	Yes (*n* = 98)		No (*n* = 320)	Yes (*n* = 118)	*p*-value
Demographic characteristics						
Site			0.07			**<0.01**
Winnipeg	64 (18.8)	9 (9.2)		63 (19.7)	10 (8.5)	
Toronto/London	53 (15.6)	19 (19.4)		41 (12.8)	31 (26.3)	
Quebec/Saguenay	223 (65.6)	70 (71.4)		216 (67.5)	77 (65.3)	
Sex assigned at birth			0.51			0.37
Female	35 (10.4)	13 (13.4)		32 (10.1)	16 (13.7)	
Male	303 (89.6)	84 (86.6)		286 (89.9)	101 (86.3)	
Age			0.85			0.38
Mean (SD)	47.75 (9.23)	47.56 (8.34)		47.48 (9.14)	48.33 (8.73)	
Service status			0.18			0.1
Active (CAF or RCMP)	39 (11.5)	6 (6.2)		38 (11.9)	7 (6)	
Veteran (CAF or RCMP)	300 (88.5)	91 (93.8)		281 (88.1)	110 (94)	
PTSD clinical characteristics						
Years of PTSD						
Mean (SD)	7.58 (5.1)	10.11 (5.71)	**<0.01**	7.43 (4.86)	10.11 (5.85)	**<0.01**
Years of pharmacological therapy			**<0.01**			**<0.01**
< 5	142 (41.8)	22 (22.4)		137 (42.8)	27 (22.9)	
5–10	114 (33.5)	41 (41.8)		109 (34.1)	46 (39.0)	
> 10	84 (24.7)	35 (35.7)		74 (23.1)	45 (38.1)	
PTSD specifier			0.11			0.35
None	295 (87.5)	79 (81.4)		275 (86.8)	99 (84.6)	
Dissociative	31 (9.2)	16 (16.5)		31 (9.8)	16 (13.7)	
Delayed expression	11 (3.3)	2 (2.1)		11 (3.5)	2 (1.7)	
Current psychotherapy use	208 (61.4)	56 (57.1)	0.53	195 (61.1)	69 (58.5)	0.69
Psychiatrist-rated confidence that psychotherapy is evidence-based 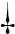	138 (66.3)	34 (60.7)	0.5	128 (65.6)	44 (63.8)	0.85
Patient comorbidities						
Any comorbid mental health conditions	265 (78.2)	78 (79.6)	0.87	247 (77.4)	96 (81.4)	0.45
Any comorbid physical health conditions	296 (87.6)	88 (90.7)	0.5	278 (87.4)	106 (90.6)	0.46

^†^Benzodiazepine prescribing was evaluated using two binary comparisons: prescriptions indicated for PTSD (yes/no) and prescriptions for any condition (yes/no); p-values correspond to between-comparison group differences. 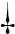
Among patients receiving psychotherapy.

PTSD – Post-traumatic stress disorder, SD – Standard deviation, IQR – interquartile range, PRN – prescribed as needed, CAF – Canadian Armed Forces.

Number of missing: Sex – 3, service status – 2; PTSD specifier – 4, comorbid mental health conditions – 1; comorbid physical health conditions – 3, current psychotherapy – 1.

Patients receiving benzodiazepines for PTSD had longer PTSD duration (mean = 10.1 years, SD = 5.7) than those not prescribed benzodiazepines (7.6 years, SD = 5.1; *p* < 0.01); a similar pattern was observed when comparing benzodiazepine prescriptions for any indication (10.1 years, SD = 5.9 vs 7.4 years, SD = 4.9; *p* < 0.01). Duration of pharmacotherapy followed a corresponding trend: among patients prescribed benzodiazepines, 78% (for PTSD) and 77% (for any indication) had been receiving pharmacotherapy for PTSD for ≥ 5 years, compared with 58% and 57% among those not prescribed benzodiazepines for PTSD or for any indication, respectively (*p* < 0.01).

### Provider-level factors among patients with and without benzodiazepine prescriptions

**Table 2** summarizes provider characteristics and the factors providers rated as important when prescribing pharmacotherapy for PTSD. Provider characteristics were linked to their respective patients, allowing comparison between those who received benzodiazepines and those who did not. Years of practice did not differ meaningfully across groups.

**Table 2:** Provider characteristics and decision-making factors by benzodiazepine prescription status.

Provider characteristic or decision-making factor	Benzodiazepines prescribed for PTSD			Benzodiazepines prescribed for any indication		
	No (*n* = 340)	Yes (*n* = 98)	*p*-value	No (*n* = 320)	Yes (*n* = 118)	*p*-value
Years of practice as licensed psychiatrist	19.14 (10.36)	20.57 (9.09)	0.22	19.07 (10.5)	20.53 (8.86)	0.18
Strategies used to assess PTSD						
Standardized clinical tools	165 (48.5)	37 (37.8)	0.08	146 (45.6)	56 (47.5)	0.82
Clinical interviews	321 (94.4)	93 (94.9)	0.99	301 (94.1)	113 (95.8)	0.65
Self-report measures	205 (60.3)	39 (39.8)	**<0.01**	187 (58.4)	57 (48.3)	0.07
Factor important* when prescribing drugs for treatment for current caseload of patients with PTSD						
Patient medication adherence	340 (100)	98 (100)	**-–**	320 (100)	118 (100)	**-–**
Positive prior experience with specific drug	340 (100)	98 (100)	**-–**	320 (100)	118 (100)	**-–**
Tolerability/avoidance of effects of drugs	340 (100)	98 (100)	**-–**	320 (100)	118 (100)	**-–**
Interactions with current medications	340 (100)	98 (100)	**-–**	320 (100)	118 (100)	**-–**
Presence of comorbid conditions	340 (100)	98 (100)	**-–**	320 (100)	118 (100)	**-–**
Patient’s prior response to medication	340 (100)	98 (100)	**-–**	320 (100)	118 (100)	**-–**
Patient’s insurance coverage	241 (70.9)	77 (78.6)	0.17	232 (72.5)	86 (72.9)	0.99
Discontinuation syndrome	236 (69.4)	77 (78.6)	0.11	228 (71.2)	85 (72.0)	0.97
Access to evidence-based psychotherapy	194 (57.1)	74 (75.5)	**<0.01**	190 (59.4)	78 (66.1)	0.24
Difficulties accessing drug in clinic/region	202 (59.4)	60 (61.2)	0.84	198 (61.9)	64 (54.2)	0.18
Provincial formulary/accessibility	189 (55.6)	53 (54.1)	0.88	183 (57.2)	59 (50.0)	0.22
Patient diet/lifestyle	171 (50.3)	38 (38.8)	0.06	164 (51.2)	45 (38.1)	**0.02**
Patient request	195 (57.4)	36 (36.7)	**<0.01**	179 (55.9)	52 (44.1)	**0.04**

*Providers rated item as important if responded “Somewhat important” or “Very important”.

PTSD, Posttraumatic stress disorder; SD, Standard deviation; IQR, interquartile range; PRN, prescribed as needed; CAF, Canadian Armed Forces.

Bold values indicate statistically significant p-values (p < 0.05).

Overall, providers across groups endorsed similar considerations when prescribing medications for PTSD. However, patients prescribed benzodiazepines for PTSD were more likely to have providers who identified access to evidence-based psychotherapy as an important consideration (76% vs 57%; *p* < 0.01). Conversely, patients prescribed benzodiazepines for PTSD were less likely to have providers who endorsed patient request as an important prescribing factor (37% vs 57%; *p* < 0.01), a pattern that was also observed when comparing prescriptions for any indication vs no benzodiazepine prescription.

### Distribution of benzodiazepine agents prescribed and prescribing modality (PRN vs scheduled)

**Supplementary Table S1** summarizes benzodiazepine prescribing patterns by agent, clinical indication, and modality of use. Lorazepam was the most prescribed agent (*n* = 70); the majority of prescriptions were for PTSD (81%), and nearly all were written on a PRN (“as needed”) basis (97%) rather than scheduled administration. Clonazepam was the second most frequently prescribed agent (*n* = 45), with approximately half prescribed PRN and the remainder scheduled; most clonazepam prescriptions were for PTSD (77%). Temazepam and diazepam were prescribed infrequently (*n* = 5–6 patients each) but, when used, were exclusively for PTSD and more often scheduled than PRN. Concurrent prescribing of more than one benzodiazepine was uncommon: 12 patients (2.7%) received two agents, most often clonazepam with lorazepam (*n* = 7). Among these patients, seven received one scheduled and one PRN benzodiazepine, while five received two PRN agents. No patient received more than two benzodiazepines concurrently or more than one scheduled benzodiazepine.

### Number of medications prescribed for PTSD and for any indication

Patients prescribed benzodiazepines for PTSD overall received a greater number of PTSD-related medications than those not prescribed benzodiazepines (mean = 4.5, SD = 1.8 vs 3.4, SD = 1.7; *p* < 0.01). Corresponding counts and proportions are presented in **Supplementary Table S2**.

**Supplementary Figure S1** illustrates the distribution of non-benzodiazepine medications co-prescribed with benzodiazepines for PTSD. Most patients receiving benzodiazepines were co-prescribed two or more non-benzodiazepine agents (84% for PTSD), and few received benzodiazepines alone (2%). **Supplementary Table S3** provides detailed counts and proportions.

### Non-benzodiazepine medication classes and individual agents co-prescribed with benzodiazepines

**Figure 1** summarizes non-benzodiazepine medication classes co-prescribed among patients treated for PTSD who were and were not prescribed benzodiazepines. Antidepressants were the most frequently prescribed class in both groups (87% among those prescribed benzodiazepines vs 88% among those not prescribed), followed by hypnotics (64% vs 60%, respectively). Within the antidepressant class, SSRIs, SNRIs, and atypical antidepressants were most common, with similar distributions across groups (SSRIs: 32% vs 37%; SNRIs: 36% vs 32%; atypical antidepressants: 33% vs 29%; **Supplementary Table S4**).

**Figure 1:**
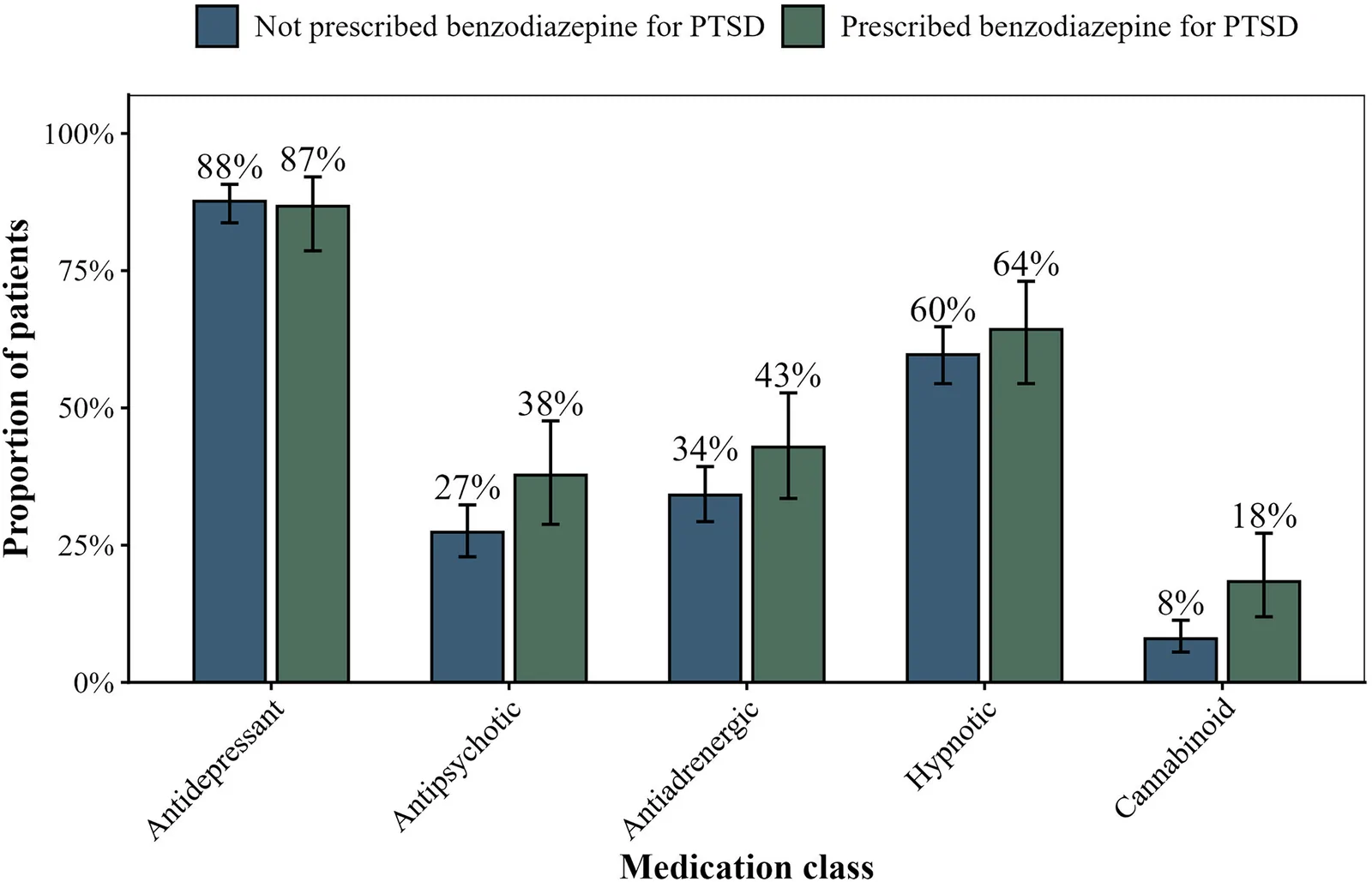
Comparison of non-benzodiazepine medication prescriptions among patients prescribed benzodiazepines for PTSD. The most frequently co-prescribed medication classes were antidepressants (87% among those on benzodiazepines and 88% among those not on benzodiazepines), followed by hypnotics (64% and 60%, respectively). Error bars represent Wilson 95% confidence intervals for each proportion.

**Figure 2** displays the most frequently prescribed individual non-benzodiazepine agents. Among patients prescribed benzodiazepines for PTSD, trazodone was the most commonly co-prescribed agent (29%), followed by zopiclone (24%) and quetiapine (24%). Among patients prescribed benzodiazepines for any indication, trazodone remained the most frequently co-prescribed agent (26%), followed by zopiclone (25%) and bupropion (22%).

**Figure 2:**
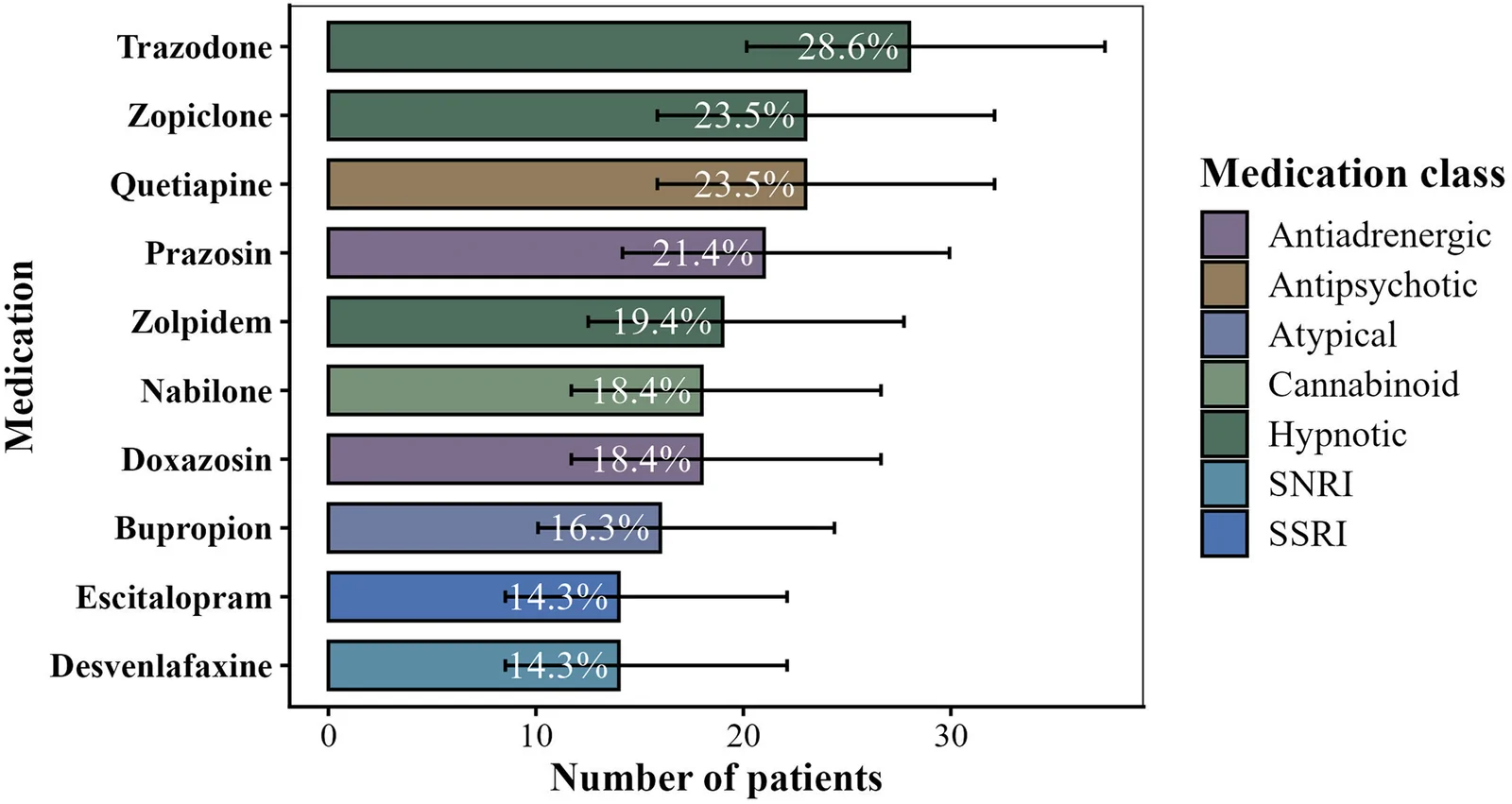
Top 10 medications co-prescribed to patients with benzodiazepines for PTSD. Trazodone was the most commonly co-prescribed medication (29% of benzodiazepine-treated patients), followed by zopiclone (24%) and quetiapine (24%). Error bars represent Wilson 95% confidence intervals for each proportion.

### Odds ratios for benzodiazepine prescribing for PTSD and for any condition

**Table 3** reports crude and adjusted odds ratios for benzodiazepine prescribing. Both longer PTSD illness and pharmacotherapy duration were significantly associated with increased odds of prescribing, and these associations remained after adjustment for clinical site, age, and sex. Models estimated with and without provider-level random effects yielded comparable results. Each additional year of PTSD duration was associated with higher odds of benzodiazepine prescribing for PTSD (adjusted OR, 1.12; 95% CI, 1.06–1.18) and for any indication (adjusted OR, 1.10; 95% CI, 1.05–1.15).

**Table 3:** Crude and adjusted odds ratios for benzodiazepine prescribing, accounting for provider-level random effects.

Predictor	Prescribed for PTSD		Prescribed for any indication	
	Crude OR	Adjusted OR	Crude OR	Adjusted OR
	(95% CI)	(95% CI)*	(95% CI)	(95% CI)*
Age	1.00 (0.97, 1.02)	-–	1.01 (0.98, 1.03)	-–
Sex				
Male	0.62 (0.3, 1.29)	-–	0.57 (0.28, 1.14)	-–
Female	REF		REF	
Site		-–		-–
Toronto/London	1.61 (0.37, 6.96)		4.73 (1.91, 11.74)**	
Quebec/Saguenay	2.19 (0.65, 7.42)		2.26 (1.02, 5.03)**	
Winnipeg	REF		REF	
Benefit status				
Veteran	1.31 (0.44, 3.84)	1.11 (0.32, 3.85)	1.61 (0.6, 4.3)	1.20 (0.37, 3.85)
Active duty	REF	REF	REF	REF
Years of PTSD	1.10 (1.05, 1.16)**	1.12 (1.06, 1.18)**	1.10 (1.05, 1.15)**	1.10 (1.05, 1.15)**
Any physical comorbidities	1.54 (0.69, 3.41)	1.61 (0.71, 3.66)	1.47 (0.7, 3.05)	1.38 (0.65, 2.93)
Any mental health comorbidities	1.16 (0.63, 2.11)	1.14 (0.62, 2.11)	1.29 (0.73, 2.28)	1.13 (0.63, 2)
Years of pharmacological therapy				
< 5	REF	REF	REF	REF
5–10	2.56 (1.39, 4.71)**	2.51 (1.34, 4.68)**	2.23 (1.27, 3.90)**	2.10 (1.18, 3.74)**
> 10	3.07 (1.58, 5.96)**	3.20 (1.59, 6.41)**	3.07 (1.69, 5.58)**	2.92 (1.57, 5.45)**
Patient receiving psychotherapy	0.93 (0.57, 1.52)	0.97 (0.58, 1.6)	0.93 (0.59, 1.46)	0.86 (0.53, 1.38)
Confidence in patients receiving EB psychotherapy	0.70 (0.35, 1.39)	0.70 (0.34, 1.41)	0.76 (0.4, 1.46)	0.72 (0.37, 1.37)

*Adjusted for clinical site and patient age and sex.

**Statistically significant (*p* < 0.05).

PTSD, Post-traumatic stress disorder; OR, Odds ratio; CI, Confidence interval.

Similarly, longer duration of pharmacotherapy was associated with increased odds of benzodiazepine prescribing. Compared with patients receiving < 5 years of pharmacotherapy, those treated for 5–10 years had higher odds of benzodiazepine prescribing for PTSD (adjusted OR, 2.51; 95% CI, 1.34–4.68) and for any indication (adjusted OR, 2.1; 95% CI, 1.18–3.74). Patients treated for >10 years had further increased odds (PTSD: adjusted OR, 3.2; 95% CI, 1.59–6.41; any indication: adjusted OR, 2.92; 95% CI, 1.57–5.45).

## Discussion

This study provides a quantitative description of benzodiazepine prescribing among Canadian military personnel and Veterans receiving specialty care for PTSD. Consistent with prior studies, benzodiazepine use was concentrated among patients with chronic PTSD, extensive treatment histories, and greater cumulative exposure to psychotropic medications (26, 41, 42). PTSD-specific prescribing showed no significant site-level variation, and accounting for provider clustering did not meaningfully change the findings.

Among the 438 patients in this cohort, the 22% prevalence of PTSD-specific benzodiazepine prescribing falls broadly within other estimates from military and civilian PTSD populations (32, 33, 35), exceeds recent VHA estimates (31, 41), and is lower than the rate reported in an acute Veteran psychiatric setting (34). Direct comparisons, however, are limited by differences in prescribing era and policy, clinical setting, PTSD severity and chronicity, and cohort and exposure ascertainment. VHA estimates, for example, derive from broad health-system cohorts identified through PTSD-coded inpatient or repeated outpatient encounters across medical centres and clinics, rather than from a tertiary-care population. Also, benzodiazepine exposure was ascertained from VHA outpatient pharmacy records alone, which the authors note may underestimate prescribing from non-VHA sources (31). In contrast, our cohort comprised patients with a primary clinical diagnosis of PTSD receiving specialist psychiatric care in tertiary OSI clinics and was likely enriched for chronic, clinically complex illness. Canadian comparisons are particularly limited. The available Quebec study examined older adults in general medical care with probable post-traumatic stress syndrome rather than diagnosed PTSD (35).

Benzodiazepines were rarely prescribed in isolation. Patients receiving benzodiazepines, who also tended to have more chronic PTSD, were prescribed a mean of 4.5 PTSD-related medications compared with 3.4 among non-recipients; 84% also received at least two additional non-benzodiazepine medications. This pattern is consistent with broader evidence that psychotropic polypharmacy in military and Veteran populations is concentrated among individuals with PTSD and greater clinical complexity. A recent meta-analysis estimated a pooled polypharmacy prevalence of 36% (95% CI, 23–49%), including 48% among Veterans with PTSD compared with 22% among those without (43). Similarly, among 1,210 Australian Defence Force Veterans transitioning to civilian life, psychotropic polypharmacy was reported in 25.9% of those with PTSD versus 4.5% of those without, and PTSD was independently associated with approximately twofold higher odds of psychotropic polypharmacy (OR, 2.07; 95% CI, 1.20–3.58) (44). Medication burden appears greater still in more acute psychiatric settings; among Australian Veterans admitted for PTSD treatment, 79.9% met criteria for psychotropic polypharmacy and 75.3% for sedative polypharmacy on admission (34). Recent national VHA data similarly demonstrate clustering of complex medication regimens, including guideline-nonconcordant prescribing, among Veterans with greater psychiatric burden (45).

Several features of military-related PTSD may contribute to the chronicity and treatment complexity represented in tertiary care. Military service can involve repeated or cumulative occupational trauma, and PTSD commonly co-occurs with psychiatric and physical conditions. In a nationally representative 16-year study of CAF personnel and Veterans, persistent or recurrent PTSD was associated with greater cumulative and deployment-related trauma exposure, subsequent trauma, and greater use of avoidance coping (46). Chronic pain is also common, although its prevalence and presentation appear to vary by PTSD course (47). A meta-analysis found smaller average symptom improvements and lower remission rates following first-line psychotherapy in military than civilian samples, despite meaningful benefit in both (48). These factors may contribute to a treatment-seeking subgroup with persistent symptoms, multimorbidity, and substantial prior treatment exposure.

### Clinical and practice implications

In clinical practice, benzodiazepine prescribing for PTSD occurs at different stages of treatment. Benzodiazepines may be introduced as a short-term adjunct for acute anxiety or insomnia during antidepressant initiation or used intermittently for situational symptoms or discrete triggers. Alternatively, they may be prescribed later when symptoms persist following guideline-concordant treatments.

Across these stages, benzodiazepines are seldom used alone and are generally prescribed alongside other agents, with ongoing reassessment and an eye toward tapering when risks begin to outweigh benefits. In our cohort, fewer than 3% of patients received more than one benzodiazepine concurrently, most often a scheduled agent combined with a PRN agent. This pattern may reflect different symptom targets or the management of breakthrough symptoms, but the available data cannot establish the clinical rationale. Complex regimens warrant periodic review of ongoing indications, potential interactions, cumulative sedative burden, and opportunities for safe deprescribing. Prescribing decisions are also shaped by a limited range of effective pharmacologic options, uneven access to psychotherapy, and practical concerns such as stigma or the consequences of abrupt discontinuation (25, 26, 49). None of these considerations diminish the well-recognized risks of benzodiazepines, but they help explain their continued, selective use in patients with chronic, treatment-experienced PTSD.

Within this context, our findings support that benzodiazepine prescribing in PTSD does not necessarily reflect poor-quality care, but more often represents a response to complex patients with persistent symptoms, including insomnia, hyperarousal, and distress, and limited remaining options. This does not render benzodiazepines benign or support routine use; rather, it suggests that strictly categorical or moral framing of their use may be clinically unhelpful.

Decisions regarding discontinuation are similarly complex. Observational work, including that of Maust et al. (50), has raised concerns about adverse outcomes associated with abrupt discontinuation of long-term benzodiazepine therapy in some populations. Current guidance emphasizes individualized, gradual tapering when the balance shifts against continued use (51). Taken together, these considerations support a more measured approach. If and when benzodiazepines are used in chronic PTSD, both continuation and discontinuation require careful judgment and ongoing monitoring.

### Limitations and study implications

This study was intended to inform a more nuanced understanding of benzodiazepine use in military-related PTSD and to guide further investigation. Findings should be interpreted in light of several limitations. The cross-sectional design provides a snapshot of prescribing and does not permit inference about temporality or causality. Our binary measure of benzodiazepine use did not distinguish intermittent from sustained use. Although the prescribed dose of each medication was recorded, it reflected only one time point. Because most benzodiazepine prescriptions were PRN and administration frequency was not captured, actual and cumulative exposure could not be estimated. We also could not characterize treatment duration, dose trajectories, adherence, clinical benefits, adverse effects, or functional change. PTSD severity, detailed comorbidity burden, and the adequacy, duration, and sequencing of prior antidepressant trials were not assessed. Prescribing intent was clinician-reported and limited to broad indication categories, which may not fully reflect how or why medications were used in practice. These limitations constrain interpretation of the timing, clinical context, and outcomes of benzodiazepine prescribing.

Future research should link longitudinal dispensing data with clinical outcomes to address these gaps. The clinician-reported approach used here could also be applied to opioids and other high-risk substances, including concurrent use with benzodiazepines, to examine prescribing indications, patterns of use, and associated outcomes in military and Veteran populations.

## Conclusion

Clinical guidelines that discourage benzodiazepine use in PTSD are appropriate at the population level, but may be insufficient when translated into the management of patients with chronic, treatment-resistant illness. In this study, prescribing in military-related PTSD was relatively uncommon and concentrated among patients with longstanding illness and substantial prior pharmacologic exposure. These findings point to an important clinical question: under what conditions, for whom, and with what safeguards should benzodiazepines be used? Addressing this will require high-quality longitudinal evidence, along with candid, operational guidance for clinicians already navigating this reality (52).

## Data Availability

The data that support the findings of this study are available from the corresponding author upon reasonable request. Due to privacy and ethical considerations, the data will not be publicly shared. Requests to access the datasets should be directed to jenny.liu@sjhc.london.on.ca.
