# Supplementary material for: Benzodiazepine prescribing in chronic military-related PTSD: real-world patterns and correlates in specialized care
Source: Front Psychiatry. 2026 Oct 5;17:1931305. doi: 10.3389/fpsyt.2026.1931305 (PMC13677842; doi:10.3389/fpsyt.2026.1931305)
Supplement: Supplementary file 1 [file SupplementaryFile1.docx]

**Supplementary File**

| **Table S1.** Frequency of benzodiazepine prescriptions among patients with primary PTSD (*n* = 438) | | | | | | |
| --- | --- | --- | --- | --- | --- | --- |
|  | **Prescriptions for any condition** | | | **Prescriptions to manage PTSD** | | |
| **Medication**  **(generic)** | **No. prescribed⸷** | **No. (%) prescribed PRN** | **No. (%) prescribed regularly** | **No. (%)**  **prescribed for PTSD** | **No. (%) prescribed PRN** | **No. (%) prescribed regularly** |
| Lorazepam | 70 | 68 (97) | 2 (2.9) | 57 (81.4) | 55 (96.5) | 2 (3.5) |
| Clonazepam | 45 | 23 (51.1) | 22 (48.9) | 34 (77.3) | 17 (50) | 17 (50) |
| Temazepam | 5 | 2 (40) | 3 (60) | 5 (100) | 2 (40) | 3 (60) |
| Diazepam | 6 | 1 (16.7) | 5 (83.3) | 6 (100) | 1 (16.7) | 5 (83.3) |
| PRN – prescribed as needed; PTSD – Post-traumatic stress disorder  ⸷Results limited to medications prescribed to five or more patients. | | | | | | |

| **Table S2.** Number of medications prescribed for PTSD and for any indication by benzodiazepine prescribing status | | | | | | |
| --- | --- | --- | --- | --- | --- | --- |
| **Count of medications** | **Benzodiazepines prescribed for PTSD** | |  | **Benzodiazepines prescribed for any indication** | | |
|  | **No (*n* = 340)** | **Yes (*n* = 98)** | ***p*-value** | **No (*n* = 320)** | **Yes (*n* = 118)** | ***p*-value** |
| 1 | 58 (17.1) | < 5 | < 0.01 | 32 (10) | <5 | < 0.01 |
| 2 | 94 (27.6) | 14 (14.3) | 0.07 | 70 (21.9) | 12 (10.2) | < 0.01 |
| 3 | 98 (28.8) | 24 (24.5) | 0.44 | 82 (25.6) | 19 (16.1) | 0.05 |
| 4 | 45 (13.2) | 16 (16.3) | 0.41 | 61 (19.1) | 29 (24.6) | 0.26 |
| 5 | 20 (5.9) | 23 (23.5) | < 0.01 | 41 (12.8) | 28 (23.7) | < 0.01 |
| 6 | 10 (2.9) | 6 (6.1) | 0.22 | 16 (5) | 12 (10.2) | 0.08 |
| 7+ | < 5 | 14 (14.3) | < 0.01 | 15 (4.7) | 16 (13.6) | < 0.01 |
| PTSD - Post-traumatic stress disorder | | | | | | |

| **Table S3.** Number of non-benzodiazepine medications co-prescribed for PTSD and for any indication among patients prescribed benzodiazepines | | |
| --- | --- | --- |
|  |  | |
| **Count of non-benzodiazepine**  **Medications** | **Benzodiazepines for**  **PTSD *(n* = 98)** | **Benzodiazepines for**  **any indication (*n* = 118)** |
| 1 | 14 (14.3) | 12 (10.2) |
| 2 | 24 (24.5) | 21 (17.8) |
| 3 | 15 (15.3) | 27 (22.9) |
| 4 | 24 (24.5) | 28 (23.7) |
| 5 | 8 (8.2) | 13 (11) |
| 6 | 5 (5.1) | 7 (5.9) |
| 7+ | 6 (6.1) | 7 (5.9) |
| PTSD - Post-traumatic stress disorder | | |

| **Table S4.** Comparison of non-benzodiazepine medication prescriptions among patients prescribed benzodiazepines  for PTSD | | | | | | |
| --- | --- | --- | --- | --- | --- | --- |
| **Medication class** | **Benzodiazepines prescribed for PTSD** | |  | **Benzodiazepines prescribed for any indication** | | |
|  | **No (*n* = 340)** | **Yes (*n* = 98)** | ***p*-value** | **No (*n* = 320)** | **Yes (*n* = 118)** | ***p*-value** |
| Antidepressant^a^ | 298 (87.6) | 85 (86.7) | 0.95 | 290 (90.6) | 104 (88.1) | 0.56 |
| SSRI | 127 (37.4) | 31 (31.6) | 0.36 | 123 (38.4) | 39 (33.1) | 0.36 |
| SNRI | 107 (31.5) | 35 (35.7) | 0.50 | 106 (33.1) | 42 (35.6) | 0.71 |
| Atypical | 100 (29.4) | 32 (32.7) | 0.62 | 113 (35.3) | 44 (37.3) | 0.79 |
| Hypnotic | 203 (59.7) | 63 (64.3) | 0.48 | 213 (66.6) | 70 (59.3) | 0.2 |
| Antiadrenergic | 116 (34.1) | 42 (42.9) | 0.14 | 114 (35.6) | 49 (41.5) | 0.31 |
| Antipsychotic | 93 (27.4) | 37 (37.8) | 0.06 | 96 (30) | 45 (38.1) | 0.13 |
| Cannabinoid | 27 (7.9) | 18 (18.4) | 0.01 | 28 (8.8) | 18 (15.3) | 0.07 |
| *Providers rated item as important if responded “Somewhat important” or “Very important”  PTSD - Post-traumatic stress disorder; SSRI – Selective serotonin reuptake inhibitor; SNRI – Serotonin-norepinephrine reuptake inhibitor; Atypical – Antidepressant agents that do not fall within SSRI/SNRI or tricyclic classes.  ^a^Subclasses do not add up to 100 as patients may be prescribed two or more types of antidepressants. | | | | | | |

**Figure S1.** Distribution of non-benzodiazepine medications co-prescribed with benzodiazepines for PTSD. Most patients were co-prescribed two or more non-benzodiazepine medications (84% for PTSD).
